# Crystal structure of the retroviral protease‐like domain of a protozoal DNA damage‐inducible 1 protein

**DOI:** 10.1002/2211-5463.12491

**Published:** 2018-08-03

**Authors:** Sushant Kumar, Kaza Suguna

**Affiliations:** ^1^ Molecular Biophysics Unit Indian Institute of Science Bangalore India

**Keywords:** crystal structure, Ddi1, DNA damage‐inducible 1 protein, *Leishmania major*, saquinavir

## Abstract

DNA damage‐inducible 1 (Ddi1) is a multidomain protein with one of the domains being retropepsin‐like. HIV‐1 protease inhibitors were found to reduce opportunistic infections caused by pathogens like *Leishmania* and *Plasmodium*, and some of them were shown to inhibit the growth of these parasites. In *Leishmania*, Ddi1 was identified as a likely target of the inhibitors. We report the crystal structure of the retropepsin‐like domain of Ddi1 from *Leishmania major* as a dimer with clear density for the critical ‘flap’ region. We have characterized binding with one of the HIV‐1 protease inhibitors in solution using bio‐layer interferometry and by docking. Further, we have performed molecular dynamics (MD) simulation studies that show that the protein undergoes a conformational change from open to semi‐open and closed forms with the closing of the flexible flap over the active site.

**Abbreviations**
BLI: bio‐layer interferometry
Ddi1: DNA damage‐inducible 1
HIV‐1 PR: HIV‐1 protease
MD: molecular dynamics
NMA: normal mode analysis
NTA: nitrilotriacetic acid
RMSF: root mean square fluctuation
RVP: Retroviral protease‐like
UBA: ubiquitin associated
UBL: ubiquitin‐like

DNA damage‐inducible 1 (Ddi1) is a multidomain protein belonging to the ubiquitin receptor family of proteins, which are known to be involved in proteasome‐mediated proteolysis of ubiquitinated proteins 1. This protein has been found to be involved in various processes essential for the functioning of the cell. It is required for the degradation of Ho endonuclease and F‐box protein Ufo1, which are involved in cell cycle progression 2, 3. It also functions in conferring growth to *pds1* mutants at restrictive temperatures, implicating a role in checkpoint control during cell division 4. It has been found to be critical for maintaining genome integrity in mammalian cells by rescuing stalled replication forks under replication stress, by removing the replisome component C20orf43/RTF2 from the stalled forks 5. It interacts with t‐SNARE and v‐SNARE proteins, which are membrane‐associated receptors involved in vesicle docking and fusion, in a phosphorylation‐dependent manner and negatively regulates late exocytotic processes in cells 1, 6, 7.

Ddi1 consists of three major domains: an N‐terminal ubiquitin‐like (UBL) domain, a retroviral protease‐like (RVP) domain in the middle, and a C‐terminal ubiquitin‐associated (UBA) domain (Fig. 1). The UBA domain of proteins belonging to the ubiquitin receptor family binds to ubiquitinated proteins, while the UBL domain binds to the 26S proteasome, thus facilitating the transfer of ubiquitinated proteins to the proteasome for degradation 8, 9, 10. Ddi1 from *Leishmania major* (*Lm*Ddi1) has been found to cleave synthetic substrates of HIV‐1 protease (HIV‐1 PR) and cathepsin D 11. In a recent study, the transcription factor SKN‐1 was reported as a natural substrate of Ddi1 in *Caenorhabditis elegans* 12. In another study, the transcription factor Nrf1, a homolog of SKN‐1, was found to be the substrate of human Ddi2 (*h*Ddi2), a homolog of Ddi1. *h*Ddi2 cleaves the transcription factor Nrf1, making it active and allowing it to upregulate expression of the proteasome 13. In a normal cell, the proteasome constitutively degrades Nrf1. When the proteasome activity is blocked by inhibitors, *h*Ddi2 cleaves Nrf1 leading to upregulation of the proteasome, often referred to as ‘bounce back’. Therefore, attempts to use proteasome inhibitors in cancer therapy have not been very successful. In such a scenario, targeting Ddi2 along with the proteasome may prevent bounce back and enable devising of a successful strategy for treating cancer. This finding has opened up a new possibility of using Ddi2 inhibitors along with proteasome inhibitors in cancer therapy.

**Figure 1:**
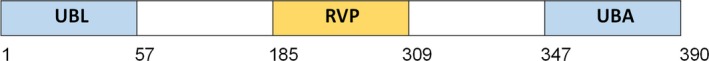
Domain architecture of Ddi1 of *Leishmania major*. RVP, retroviral protease‐like domain; UBA, ubiquitin‐associated domain; UBL, ubiquitin‐like domain.

Over the last few years, Ddi1 has attracted major attention for being a potential drug target for treating opportunistic infections. The pathogens responsible for opportunistic infections mainly belong to the trypanosomatid family, for example, *Leishmania*,* Plasmodium*,* Trypanosoma*, and *Toxoplasma*. Moreover, these parasites pose a major health challenge in developing and poor nations. About 200 000–400 000 cases of visceral leishmaniasis and 700 000–1 200 000 cases of cutaneous leishmaniasis occur every year, resulting in 20 000–40 000 deaths 14. About 300 million people are at risk of infection by visceral leishmaniasis worldwide. It was observed that in AIDS patients receiving highly active antiretroviral therapy, the treatment also offered protection against opportunistic parasitic diseases like leishmaniasis 15, 16. In the last few years, many HIV‐1 PR inhibitors have been tested on various pathogenic parasites such as *Leishmania*,* Toxoplasma*,* Trypanosoma*, and *Plasmodium* for their direct ability to inhibit growth of or kill the pathogens 17, 18, 19, 20, 21, 22, 23. One of the initial studies showed a dose‐dependent anti‐leishmanial activity of indinavir and saquinavir on *Leishmania major* with 50% lethal dose values of 8.3 and 7 μm, respectively 17. Another study reported IC_50_ values for nelfinavir and saquinavir to be 13.37 and 46.95 μm, respectively 18. Further studies on *Leishmania amazonensis* established the role of other HIV‐1 PR inhibitors in impairing parasite growth in a dose‐dependent fashion. The IC_50_ values for nelfinavir and lopinavir were found to be 15.12 and 16.47 μm, respectively, while for amprenavir it was 62 μm after 48 h of growth 19. In the same study, these protease inhibitors were also found to have a profound effect on the morphology of *Leishmania* parasites. Transmission electron microscopy revealed features that were suggestive of autophagy such as shrinking cytoplasm, an increase in the number of vesicles, and wrapping of the nucleus by endoplasmic reticulum. Nelfinavir was shown to generate oxidative stress in *Leishmania* amastigotes leading to altered physiological parameters such as an increase in the sub‐G1 DNA content, nuclear DNA fragmentation, and loss of mitochondrial potential, which are all characteristics of apoptosis 20. Fluorescence microscopy studies on *Leishmania infantum* and *Leishmania mexicana* treated with saquinavir and nelfinavir showed the presence of binucleate and, in some cases, multinucleate cells, suggesting that these inhibitors interfered with cell division 18. It was found that protease inhibitors also reduce intracellular survival of *Leishmania* species 21. Anti‐proliferative action of ritonavir and nelfinavir has also been reported on *Toxoplasma gondii*, which is responsible for toxoplasmic encephalitis 24.

Though these studies did not identify the actual target of HIV‐1 PR inhibitors, they led to the speculation that the inhibitors might target aspartic proteases in these organisms. Aspartic protease activity was detected in the soluble fraction of *L. mexicana* promastigotes by Valdivieso *et al*. 25. Ddi1 was identified as being the only active aspartic protease present in *L. major* 11 indicating the possibility of its being the target for HIV‐1 PR inhibitors. The study of White *et al*. 26 in yeast *DDI1* knockout strains confirmed that Ddi1 is the target of HIV‐1 PR inhibitors by monitoring protein secretion levels regulated by Ddi1. While this function was lost in the knockout strains, which show increased levels of protein secretion, it was restored by the introduction of *Leishmania* Ddi1, which was again inhibited by HIV‐1 PR inhibitors. In addition, mutating the active aspartate of *Leishmania* Ddi1 to alanine aborted the function. These results provided additional evidence for the RVP domain of Ddi1 (Ddi1‐RVP) to be the likely target for HIV‐1 PR inhibitors.

So far, crystal structures of Ddi1‐RVP have been reported only from *Saccharomyces cerevisiae* (*y*Ddi1‐RVP) and humans, but not from parasitic protozoans 27, 28, 29. Also, the reported structures lack electron density of either one or both of the flap regions, which are known to be important for substrate/inhibitor binding in aspartic proteases. None of the previous studies provides evidence of direct interaction between the Ddi1‐RVP and HIV‐1 PR inhibitors. Currently, many HIV protease inhibitors are commercially available. Though their detrimental effect on these protozoan parasites is now well‐established, they are not potent enough to be used directly against these pathogens as drugs. There are ample possibilities to modify these inhibitors so as to increase their specificity and toxicity to pathogens. However, limited structural information on Ddi1 in its free and inhibitor‐bound forms from any of the protozoans makes this endeavor difficult. Here, we report the first crystal structure of the RVP domain of Ddi1 from the protozoan *L. major* in two different forms that reveal the nature of the entire flap region. We performed normal mode analysis and molecular dynamics (MD) simulation studies to understand the dynamics of the protein. Further, we carried out *in vitro* binding studies with HIV‐1 PR inhibitors using bio‐layer interferometry (BLI) followed by molecular docking to understand the nature of the interaction. In this paper, we report binding of an HIV‐1 PR inhibitor directly to Ddi1 of *L. major*, which should help further explore and expand inhibitor binding studies to Ddi1 of other protozoa in an attempt to design effective drugs against many infections.

## Materials and methods

### Cloning, protein expression, and purification

The *Ddi1* gene (1170 bp) from *L. major* (Gene ID: 12983071) was synthesized (GenScript, Piscataway, NJ, USA), and three different constructs, *Lm*Ddi1_1–390_, *Lm*Ddi1_1–310_, and *Lm*Ddi1_184–390_, were subcloned in pET‐28a vector between NdeI and HindIII sites with an N‐terminal hexa‐histidine tag. Plasmids containing the gene of interest were transformed into *E. coli* BL‐21 (DE23) competent cells and plated on LB agar medium containing 30 μg·mL^−1^ kanamycin. A single colony of the transformed *Escherichia coli* BL‐21 cell was taken and inoculated in LB containing 30 μg·mL^−1^ kanamycin and grown at 37 °C for 10–12 h. One per cent of the overnight culture was inoculated in the LB and grown at 37 °C until the optical density at 600 nm reached around 0.6. The protein expression was induced by adding 0.20 mm isopropyl β‐d‐1‐thiogalactopyranoside, and the cells were further grown at 18 °C for 16–20 h. The cells were harvested by centrifugation at 7000 ***g*** for 10 min. The pellet was resuspended in a lysis buffer containing 20 mm Tris/HCl (pH 7.5), 300 mm NaCl, and 2 mm β‐mercaptoethanol. The cells were lysed by sonication and the lysate subjected to centrifugation at 18 300 ***g*** for 30 min. The supernatant was passed through a column containing Ni‐nitrilotriacetic acid (NTA) beads (GE Healthcare, Uppsala, Sweden); 20 column volumes of a buffer containing 20 mm Tris/HCl (pH 7.5), 300 mm NaCl, 2 mm β‐mercaptoethanol, and 10 mm imidazole were passed through the column to remove impurities bound non‐specifically to the column. The protein was eluted with an elution buffer containing 20 mm Tris/HCl (pH 7.5), 300 mm NaCl, and 300 mm imidazole. A final round of purification was carried out by gel filtration chromatography using an S‐200 Superdex (GE Healthcare) preparatory column equilibrated with buffer containing 20 mm Tris/HCl (pH 7.5), 100 mm NaCl, and 2 mm β‐mercaptoethanol on Bio‐Rad (Hercules, CA, USA) Duoflow fast protein liquid chromatography system at a flow rate of 1.00 mL·min^−1^. The purity of the protein was checked by running 12% SDS/PAGE. The mass of the protein was confirmed by matrix‐assisted laser desorption/ionization time of flight mass spectrometry.

### Bio‐layer interferometry

Binding of HIV protease inhibitors with *Lm*Ddi1_1–390_ was measured by BLI using an Octet Red96 system (Pall FortéBio, Fremont, CA, USA). The protein was immobilized on the Ni‐NTA sensor tip by dipping the sensor tip in a 10 μm protein solution for 5 min. The protein‐bound sensor tip was then dipped in a solution containing 20 mm MES buffer (pH 6.0), 200 mm NaCl, and 1% DMSO for 10 min to remove non‐specifically bound protein. The sensor tip was sequentially dipped in the solution containing 20 mm MES buffer (pH 6.0), 200 mm NaCl, 1% DMSO, and HIV‐1 PR inhibitor (saquinavir, nelfinavir, lopinavir, and amprenavir) at 75, 150, 300, and 600 μm concentrations. The traces were processed using fortebio octet data analysis software, v. 8.0.3.5 (Pall FortéBio), exported, and fit globally. A simple 1 : 1 Langmuir interaction model was used for fitting the data. Double referencing was performed to avoid effects of any non‐specific interaction of the inhibitor with the sensor tip or with the protein.

### Crystallization

Crystallization was carried out by microbatch method with 2 μL each of the protein and precipitant solution. The protein solution at a concentration of 7–12 mg·mL^−1^ was mixed with various precipitants (Hampton Research, Aliso Viejo, CA, USA) and placed in the well of the microbatch plate layered with paraffin and silicon oil (1 : 1) at 291 K. Crystals for the construct *Lm*Ddi1_1–390_ were obtained in a condition containing 0.1 m sodium malonate pH 4.0 and 12% w/v polyethylene glycol 3350 after 2 weeks. Crystals for the construct *Lm*Ddi1_184–390_ were obtained in a condition with 0.1 m sodium acetate trihydrate pH 4.5, 30% w/v polyethylene glycol 1500 after 3 weeks.

### Data collection and structure determination

X‐ray diffraction data for the crystal of the construct *Lm*Ddi1_1–390_ were collected at the European Synchrotron Radiation Facility (ESRF) BM‐14, Grenoble; 520 frames were collected at a wavelength of 0.976 Å at 100 K with an oscillation angle of 0.5° per image and a crystal to detector distance of 150 mm using a Marmosaic 225 CCD detector. Data for the crystal of the construct *Lm*Ddi1_184–390_ were collected at the home source at 100 K, with Cu Kα radiation generated by a Rigaku Micromax‐007HF X‐ray generator and focused by the Osmic mirror optics system; 154 frames were recorded using a mar research imaging plate (MAR345) detector system with an oscillation angle of 1° per image with a crystal to detector distance of 180 mm. The data were processed and scaled using mosflm and aimless of the ccp4 suite, respectively 30. The data collection and processing statistics are given in Table 1. The structure solutions were obtained by molecular replacement using phaser 31. The structures were manually built using coot 32 starting from the solutions obtained by the phaser, and refined by refmac5 33 or phenix.refine 34. Several rounds of alternating model building and refinement cycles were carried out until the *R*‐factor and *R*_free_ converged. Water molecules were identified by automatic water‐picking algorithm of coot. The positions of these automatically picked waters were manually examined, and additional water molecules were identified on the basis of electron density map contoured at 1.0σ in the 2*F*_o_ *−F*_c_ map and 3.0σ in the *F*_o_ *−F*_c_ map. The final structures were validated using molprobity 35. All the figures were generated using UCSF chimera 36. Atomic coordinates and structure factors have been deposited in the Protein Data Bank with accession codes 5YS4 and 5YQ8 for *Lm*Ddi1‐RVP domains obtained from *Lm*Ddi1_1–390_ and *Lm*Ddi1_184–390_ constructs, respectively.

**Table 1:** Data collection and refinement statistics

	*Lm*Ddi1_184–390_ (PDB: 5YQ8)	*Lm*Ddi1_1–390_ (PDB: 5YS4)
Wavelength (Å)	1.54	0.97
Resolution range	58.55–2.25 (2.32–2.25)	27.61–2.30 (2.38–2.30)
Space group	P2_1_	P1
Unit cell		
*a*,* b*,* c* (Å)	35.53, 80.39, 85.45	43.79, 56.78, 91.40
α, β, γ (°)	90, 90, 90	76.56, 84.48, 89.99
No of molecules/asymmetric unit	4	6
Total reflections	58 190 (4981)	101 154 (9861)
Unique reflections	22 187 (2068)	36 968 (3613)
Multiplicity	2.6 (2.4)	2.7 (2.7)
Completeness (%)	96.9 (98.7)	97.6 (96.9)
Mean *I*/σ(*I*)	5.9 (2.7)	4.9 (1.8)
Wilson B‐factor (Å^2^)	14.6	26.37
*R*_merge_ (%)	13.6 (34.0)	13.0 (62.8)
*R*_pim_ (%)	13.6 (33.9)	9.3 (45.6)
CC (1/2)	0.971 (0.324)	0.981 (0.612)
*R*_work_ (%)	16.6	23.10
*R*_free_ (%)	23.8	25.52
No. of non‐hydrogen atoms		
Macromolecules	3930	5701
Solvent	488	388
RMSD (bonds) (Å)	0.0196	0.003
RMSD (angles) (°)	1.893	0.628
Residues in Ramachandran plot (%)		
Most favored region	96.21	97.92
Additionally allowed region	3.78	2.08
Outliers	0	0
Average B‐factor (Å^2^)		
Macromolecules	16.46	35.75
Solvent	15.03	34.40

Values in parentheses are for the highest resolution shell.

### Molecular dynamics simulation

MD simulation was carried out using the gromacs package version 5.0.4 with amber 99SB force field 37. The protein was solvated using the TIP3P water model 38 in a dodecahedron box. A minimum distance of 1.0 nm was kept between the protein and the box walls. A concentration of 0.1 m NaCl was added to the system to mimic more closely the physiological condition keeping the system neutral. Long range electrostatics interactions were calculated with the particle mesh ewald (PME) method 39. Distance cutoff for van der Waals interaction was kept at 10 Å. Bonds were constrained using the lincs algorithm 40. Energy minimization was carried out using steepest descent method. The system was equilibrated at NVT ensemble followed by NPT ensemble for 100 ps each keeping the position of atoms restrained by applying a force constant of 1000 kJ·mol^−1^·nm^−2^ on protein atoms. Temperature equilibration was performed using a modified Berendsen thermostat 41 with a coupling time constant of 0.1 ps and a reference temperature of 300 K. Pressure equilibration was performed using the Parrinello–Rahman method 42 with a coupling time constant of 2 ps and reference pressure of 1 bar. A time step of 2 fs was used in the leap‐frog integrator. Coordinates and energies were written every 10 ps. Analysis of the simulation trajectories was carried out using tools available in the gromacs package. vmd was used for the visualization of trajectories 43.

### Molecular docking

Molecular docking of *Lm*Ddi1‐RVP with saquinavir was carried out using autodock vina 44. Saquinavir structure was extracted from the crystal structure of the HIV‐1 saquinavir complex (PDB Code: 3N3I). Molecules were prepared for docking using autodock tools version 1.5.6 45.

Structure‐based sequence alignment was carried out using the webserver ESPript3 46. Electrostatic surface potential was generated using PDB2PQR server 47.

## Results

Three different constructs of Ddi1 from *L. major* were used in our study: *Lm*Ddi1_1–390_ containing all three domains, *Lm*Ddi1_1–310_ comprising the UBL and the RVP domains, and *Lm*Ddi1_184–390_ having the RVP and the UBA domains. Crystals were obtained for the constructs *Lm*Ddi1_1–390_ and *Lm*Ddi1_184–390_.

Crystals obtained from the construct *Lm*Ddi1_1–390_ are in the P1 space group. The structure was determined by molecular replacement using the *y*Ddi1‐RVP structure (PDB Code: 2I1A) as the search model. In the electron density, we could trace the chain corresponding to the RVP domain only with three dimers in the asymmetric unit, suggesting that the rest of the protein was cleaved and did not crystallize. Electron density of the entire RVP domain (*Lm*Ddi1‐RVP) from amino acid residues 184 to 310 is present except for residues 230–240. The sequence of HIV‐1 PR corresponding to the missing residues of *Lm*Ddi1‐RVP is known to form a glycine‐rich β‐hairpin structure referred to as the ‘flap’, an important structural determinant in substrate and inhibitor binding.

The other construct, *Lm*Ddi1_184–390_, crystallized in a monoclinic system. The structure solution could be obtained in space group P2_1_ by molecular replacement, using *Lm*Ddi1‐RVP dimer of the P1 space group, with translation function *Z*‐score and log‐likelihood gain values of 68 and 6634, respectively. The asymmetric unit has two dimers of the RVP domain only related by an approximate twofold non‐crystallographic symmetry. Clear density appeared for both the flaps (Met231–Arg246) of the dimers suggesting that they are well ordered, unlike those in the P1 space group. The flaps seem to be ordered due to the restricted movement imposed by crystal packing. Both the dimers in P2_1_ are identical with small differences only in the flap regions as discussed in the next section.

### Structure of *Lm*Ddi1‐RVP

**Figure 2:**
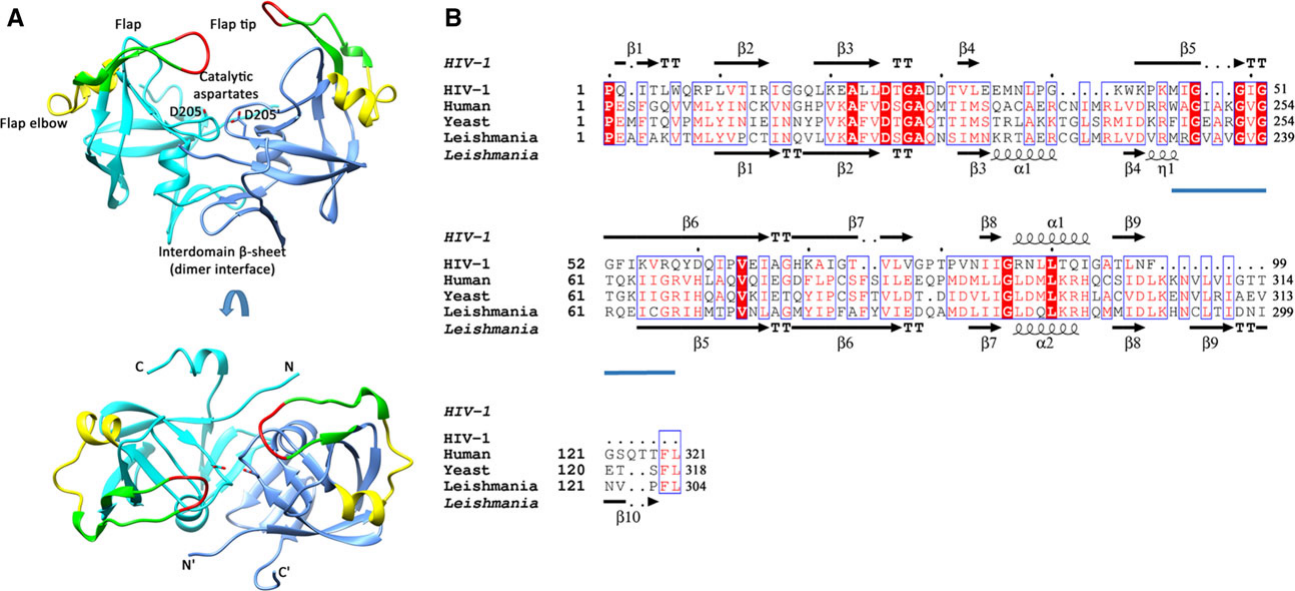
Crystal structure of *Lm*Ddi1‐RVP and sequence comparison. (A) The dimeric structure of *Lm*Ddi1‐RVP showing the flap in green (residues 231–246), tip of the flap in red (residues 237–240), and flap elbow in yellow (residues 215–228). Catalytic aspartates are shown as sticks. N‐ and C‐termini of each subunit are labeled. (B) Structure‐based sequence alignment of *Lm*Ddi1‐RVP with HIV‐1 protease (PDB: 4HVP) showing their secondary structural elements. Flap region is underlined in blue.

**Figure 3:**
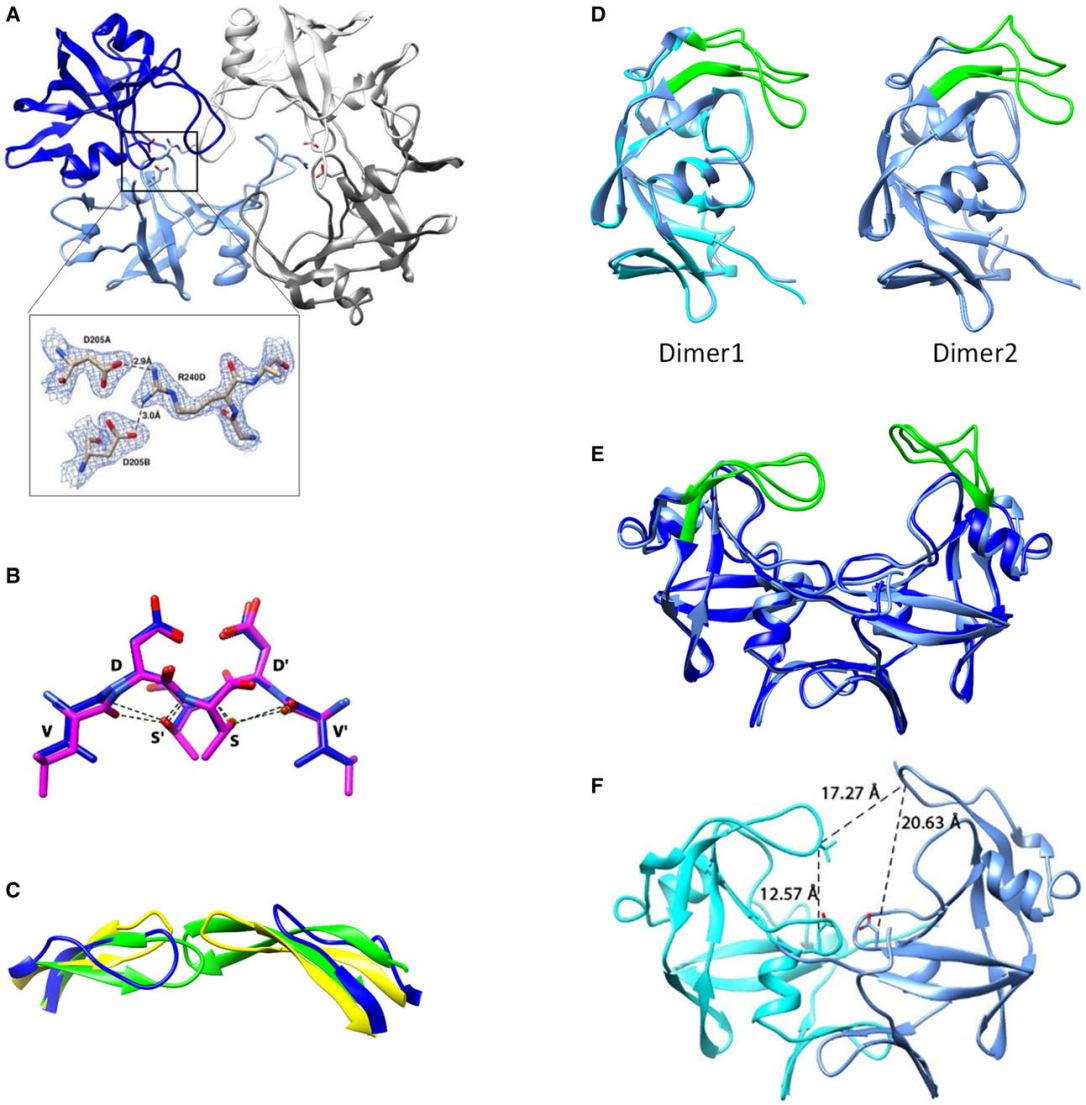
Structural details of *Lm*Ddi1‐RVP and comparison with HIV‐1 PR structure. (A) The structure of *Lm*Ddi1‐RVP showing interaction of catalytic aspartates with flap arginine from neighboring dimer. Enlarged view of interacting residues with the 2*F*_o_−*F*_c_ electron density contoured at 1σ. (B) Fireman's grip of *Lm*Ddi1‐RVP and HIV‐1 PR shown in purple and blue, respectively. (C) Superimposition of HIV‐1 PR flaps in its open (yellow) and closed conformation (green) over the flap of *Lm*Ddi1‐RVP (blue). (D,E) Superposition of two subunits in each dimer (D) and two dimers of the asymmetric units (E) of *Lm*Ddi1‐RVP structure showing differences in the flap conformation. (F) *Lm*Ddi1‐RVP structure showing flap distance measured between the Cα of Val238 and Val238′ and distance between the flap tip and catalytic aspartates measured between Cα of Val238 and Cβ of Asp205.

The fold and the catalytic architecture of *Lm*Ddi1‐RVP are similar to those of HIV‐1 PR. It is a homodimer where each polypeptide chain forms a β‐barrel structure and contributes one aspartate to the catalytic site at the dimer interface (Fig. 2A). In HIV‐1 PR, the region between the strands β4 and β5, consisting of amino acid residues from 35 to 42, is called the flap elbow region. This region in *Lm*Ddi1‐RVP has an insertion of five residues and an additional α‐helix between Lys215 and Arg220 (Fig. 2B). Also, there are two additional β‐strands near the C‐terminus, β9 (Cys293–Ile296) and β10 (Ile299–Pro302) compared to HIV‐1 PR (Fig. 2B). These strands, along with the preceding strand β8 (Met285–Asp288) from each chain, come together at the base of the structure forming a single continuous β‐sheet, referred to as the interdomain β‐sheet. In HIV‐1 PR, this sheet is formed by two strands, β1 at the N terminus and β9 at the C terminus, from each subunit. In *Lm*Ddi1‐RVP, the interaction between the two polypeptide chains is limited to this β‐sheet region with a buried surface area of about 2300 Å^2^. The characteristic ‘ψ loop’ containing the catalytic aspartates and the hydrophobic–hydrophobic–glycine (HHG) motif is present in the structure, with the motif being formed by residues Ile274, Ile275, and Gly276. In HIV‐1 PR, the catalytic aspartates coordinate with a water molecule, whereas the same position is occupied by the side chain of Arg240 residue from the flap of a neighboring molecule (the second dimer in the asymmetric unit) in *Lm*Ddi1‐RVP (Fig. 3A), making salt bridge interactions with the catalytic aspartates. Though this interaction is a consequence of packing of molecules in the crystal, this information can be utilized in designing peptide‐based inhibitors for the Ddi1‐RVP domain. Similar blocking of the active site was reported in cathepsin D where a lysine residue from the N‐terminal region inserts into the active site and interacts with the catalytic aspartates resulting in inactivation of the enzyme at high pH 48. A network of hydrogen bonds, often referred to as the ‘fireman's grip’ arrangement, involved in the stabilization of the active site is also conserved in *Lm*Ddi1‐RVP (Fig. 3B). Though the overall sequence similarity of the Ddi1 proteins is not very high among different organisms, the sequence of the RVP domain is highly conserved.

In HIV‐1 PR, the flap closes over the substrate/inhibitor bound to the active site. It assumes a distinct β‐hairpin structure and exists in an open or semi‐open conformation in the inhibitor‐free form, and in a closed conformation in the inhibitor‐bound form (Fig. 3C). The flap of *Lm*Ddi1‐RVP lacks the characteristic β‐hairpin structure and forms a loop‐like structure that hangs over the active site asymmetrically (Fig. 2A). The asymmetry results because the conformations of the flaps are slightly different in the two subunits in each dimer (Fig. 3D). Also, the flaps in two dimers in the asymmetric unit have different confirmations (Fig. 3E). The distance between the Cα atoms of Val238 residues located at the tip of the two flaps is 17.27 Å, similar to the open form of HIV‐1 PR (Fig. 3F). In contrast to HIV‐1 PR, the flaps are not equidistant from the active site. In one subunit, the flap lies close to the active site where the distance between the Cα atom of Val238 and the Cβ atom of Asp205 (catalytic aspartate) is 12.57 Å, whereas the same distance in the other subunit is 20.63 Å (Fig. 3F). This makes the binding pocket of *Lm*Ddi1‐RVP wider on one side. The amino acid sequence of the flap is known to affect its dynamics in retropepsins, an important determinant in binding to the inhibitors 49, 50, 51. Mutations in the flap region were shown to weaken the binding of the inhibitor 50, 52, 53. The sequence of the flap of *Lm*Ddi1‐RVP differs significantly from that of HIV‐1 PR except for the conserved glycines at positions 237 and 239 in the tip region (Fig. 2B). Differences in the flap sequence may influence its dynamics and hence its ability to bind inhibitors.

**Figure 4:**
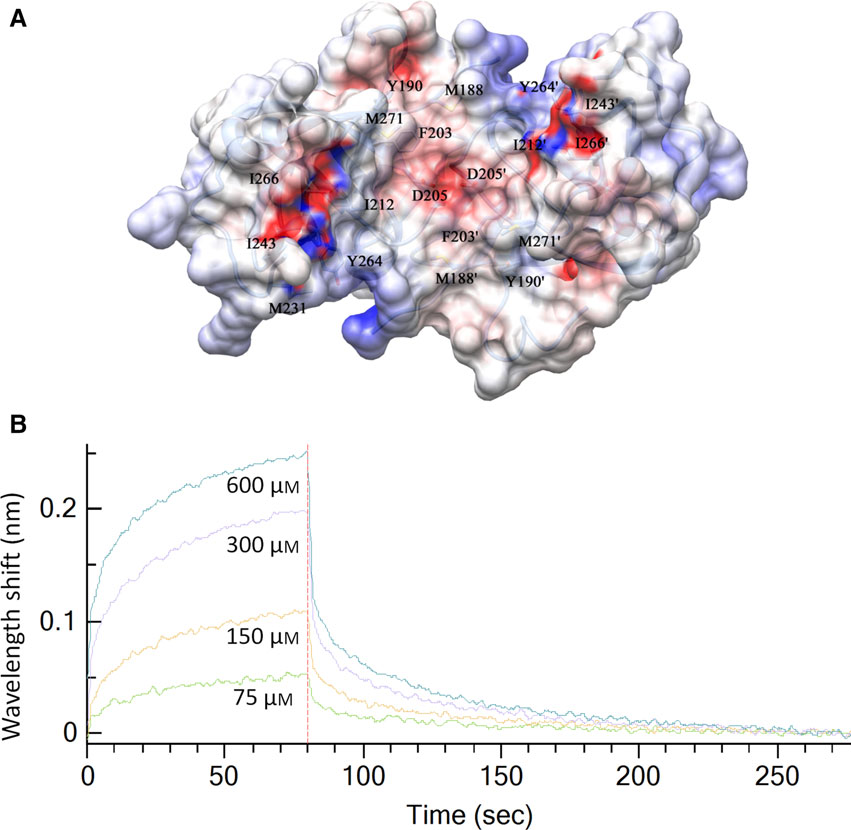
Binding pocket of *Lm*Ddi1‐RVP and its interaction with HIV‐1 PR inhibitors. (A) Surface representation of *Lm*Ddi1‐RVP showing hydrophobic binding cavity (gray) with an acidic path formed by catalytic aspartates (red). Loops have been deleted for a better representation of underlying cavity. (B) Binding plot obtained by bio‐layer interferometry showing interaction between *Lm*Ddi1‐RVP and saquinavir.

The substrate binding cavity is lined by hydrophobic amino acid residues. The side chains of Met188, Tyr190, Phe203, Ala208, Ile212, Met231, Ile243, Tyr264, Ile266, and Met271 face the substrate binding cavity forming a hydrophobic surface with an acidic patch formed by the catalytic residue Asp205 (Fig. 4A). The width of the cavity thus formed is approximately 24 Å measured between the Cα atom of Gln269 of the two subunits. The cavity of *Lm*Ddi1‐RVP is bigger than that formed in HIV‐1 PR, which has a width of approximately 19–22 Å in the open form. In HIV‐1 PR, as the dimer is symmetric, the same residues form S1 and S1′ subsites for the P1 and P1′ positions of the substrate, respectively. This is true for *Lm*Ddi1‐RVP as well where the S1/S1′ subsites are formed by Phe203 and Gly207, while in HIV‐1 PR, these positions are occupied by Leu and Gly residues, respectively. The S2/S2′ subsites are formed by Ala208, Ile212, and Ile274, which are the same as those in HIV‐PR except for a Val residue that replaces Ile212; the S3/S3′ subsites have residues Gln209, Met188, Met271, and Tyr190, while the corresponding ones are Glu, Arg, Pro, and Lys, respectively, in HIV‐PR. Therefore, S1/S1′ and S2/S2′ subsites of *Lm*Ddi1‐RVP have very similar amino acid residues to those of HIV‐1 PR and seem to have a strong preference for hydrophobic amino acid residues at both the P1 and P1′ positions like HIV‐1 PR.

### Interaction of *Lm*Ddi1 with HIV‐1 PR inhibitors

Though the inhibitory effect of HIV‐1 PR inhibitors on the growth of *L. major* has been studied, there is no evidence of the interaction of these inhibitors directly with *Lm*Ddi1. Subsequent studies indicated *Lm*Ddi1 to be the likely target of these inhibitors. In order to quantify the interaction, we carried out *in vitro* binding studies of *Lm*Ddi1_1–390_ with HIV‐1 PR inhibitors saquinavir, nelfinavir, amprenavir, and lopinavir using BLI. Of all the inhibitors tested, saquinavir showed the highest affinity for the protein. However, the binding strength of saquinavir itself was weak as shown by a high *K*_d_ value of 314 ± 12.8 μm (Fig. 4B). Nelfinavir showed much less binding, and the binding affinity could not be quantified due to a weak signal. Amprenavir and lopinavir did not show any binding with the protein.

### Normal mode analysis

**Figure 5:**
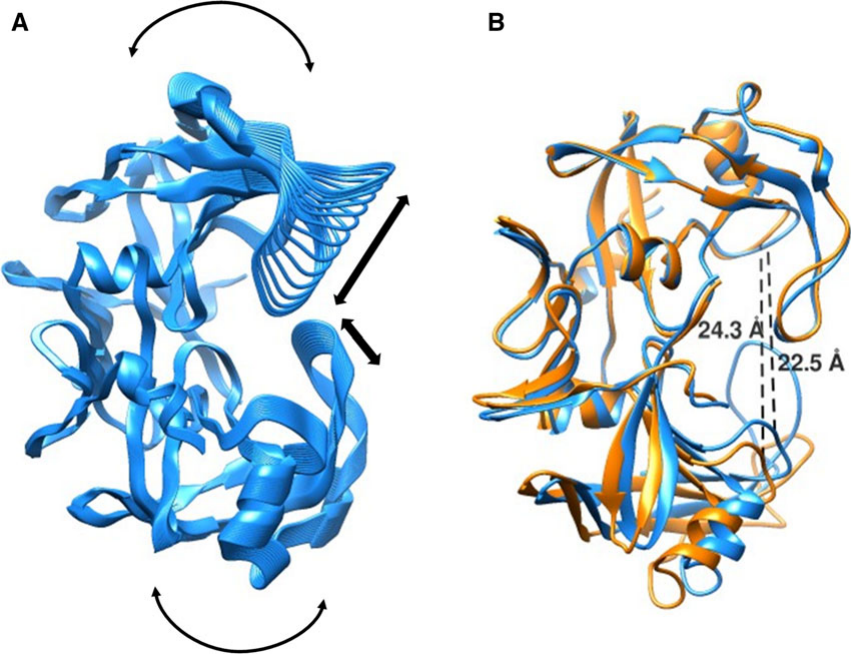
Structural fluctuations captured in normal mode analysis (NMA). (A) Direction of structural motion as revealed by NMA. (B) Superposed structure of *Lm*Ddi1‐RVP (orange) over its closed form obtained by NMA (blue) showing change in active site width measured between the C_α_ atoms of Gln269 and its symmetry‐mate.

The crystal structures of HIV‐1 PR with different inhibitors show that the flaps close upon inhibitor binding. In addition, there is a global conformational change that allows the two subunits to move closer, resulting in a tight closure of the pocket and flaps over the substrate/inhibitor. The width of the binding cavity, measured between the Cα atom of Pro81 and its symmetry‐mate, is 21.90 Å in its open form (PDB code: 3HVP), which reduces to 18.86 Å in its closed form (PDB code: 4HVP) upon inhibitor binding. To probe whether *Lm*Ddi1‐RVP can undergo similar movements, normal mode analysis (NMA) was carried out using the server, NOMAD‐Ref 54. NOMAD‐Ref uses an elastic network model to generate movements in biological macromolecules that are functionally relevant. In the analysis, a total of 16 modes were generated and analyzed. Of all the modes, mode 7 showed movements very similar to those observed in HIV‐1 PR from the open to the closed form (Fig. 5A). Also, NMA revealed that the movement extends to the rest of the protein as well. As a result, the flap closes over the active site in a way similar to that in HIV‐1 PR accompanied by a decrease in the size of the binding cavity (Fig. 5B).

### MD simulation

**Figure 6:**
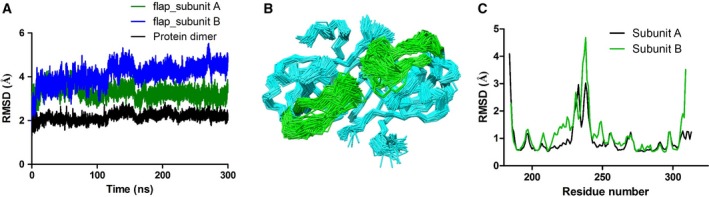
Structural fluctuation during MD simulation. (A) Plot showing RMSD values of Cα atom between the crystal structure and the structures obtained from the MD simulation. (B) Wire representation of superimposed structures generated during the simulation, depicting significant movement in the flap region (green). (C) Residue‐wise RMSF fluctuations of two subunits showing highest fluctuation in the flap region.

**Figure 7:**
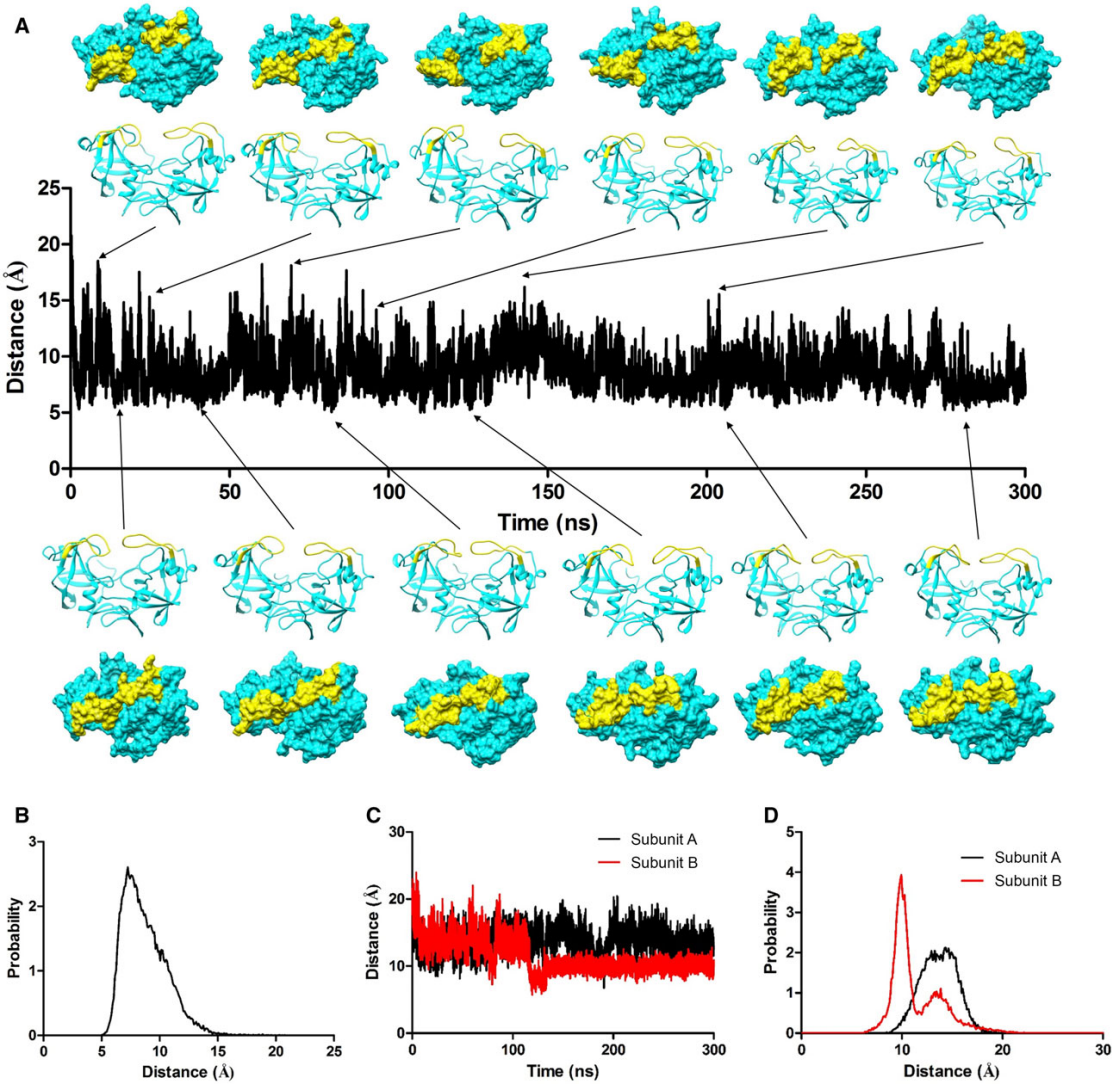
Plot showing distance between the two tips of the flaps. (A) Distance of the two tips of the flaps during the simulation measured between Cα of Val238 and Val238′. Cartoon representation of structures (side view) along the trajectory at different time points showing various closed, semi‐open, and open conformations along with the corresponding surface representation (top view) depicting access to the active site. Flap is colored yellow. Access to the open state is more frequent during the first 100 ns, and thereafter, protein prefers to stay mostly in semi‐open form. (B) corresponding histogram of flap tip distance. (C) Distance plot between Cα of Val238 and Cβ of Asp205 and (D) the corresponding histogram. Flap of subunit B comes very close to active after about 120 ns, while flap of subunit A maintains similar distance distribution throughout the simulation.

Movement and dynamics of the flap are known to play a very important role in substrate/inhibitor binding in aspartic proteases. We carried out an all‐atom molecular dynamics simulation of *Lm*Ddi1‐RVP for 300 ns to understand in detail the nature of the flap movement and its inherent flexibility. During the simulation, the protein dimer remained stable and stayed close to the crystal structure. The RMSD values of Cα atoms between the structures generated over the course of simulation and the crystal structure was about 2 Å (Fig. 6A). The flaps showed significant movement in both the subunits and assumed a wide range of conformations that vary from closed, to semi‐open, to open (Fig. 5B). The RMSD values of the flap of one subunit (subunit A) increased over the course of the simulation and stabilized at about 4.5 Å, whereas that of the flap of the other subunit (subunit B) showed a similar increase during the initial duration but eventually decreased and stabilized at about 3 Å near the end of the 300 ns run (Fig. 6A). Interestingly, during the entire run, it did not assume any ordered β‐hairpin structure. Also, the flaps showed asymmetric behavior in terms of their dynamics. In subunit B, the flap showed more flexibility compared to that of subunit A as shown in the root mean square fluctuation (RMSF) plot (Fig. 6C) and superimposed structures generated during the simulation run (Fig. 6B). During the simulation, it showed a wide range of conformations showing both horizontal and lateral movements resulting in various states between closed and open as shown in Fig. 7A. The closest distance that the two flaps come together, measured between the Cα atoms Val238 of the two flaps, is about 5 Å, while the farthest distance is about 20 Å, similar to the closed and open forms, respectively, of HIV‐1 PR (Fig. 7B). In the first 100 ns of simulation, the structure sampled the open conformation more frequently (Fig. 7A). Afterwards, for most of the time, it remains in a semi‐open conformation where the distance between the Cα atoms of the two Val238 residues is about 6.5 to 10 Å, implying that the semi‐open form is the most thermodynamically stable state, and what is observed in the crystal structure is the effect of crystal packing. The open state is a less likely event as suggested by the low probability of its occurrence. Unlike HIV‐1 PR, where flap opening is accompanied by a large movement in the elbow region, the flap movement in *Lm*Ddi1‐RVP does not significantly affect another part of the protein. This is probably due to the presence of an additional secondary structural element (α1 in Fig. 2B) in the elbow region that makes it relatively rigid. The tip of the flap of subunit A maintains a uniform distance from the catalytic aspartates showing a unimodal distribution with a most probable distance of about 13–15 Å measured between the Cα atom of Val238 and the Cβ atom of Asp205 (Fig. 7C,D). In subunit B, this distance shows a bimodal distribution where it is about 14 Å initially, but after 115 ns, the flap moves inside the binding pocket, thus decreasing the distance to about 9–10 Å. Another characteristic feature in HIV‐1 PR is the curling of the tip of the flap, which is known to trigger flap opening 55, 56, 57. The tip of the flap has a Gly‐Ile‐Gly motif that imparts it high flexibility. In *Lm*Ddi1‐RVP, this motif is Gly‐Val‐Gly, and a similar curling is seen during the simulation. The tip undergoes very rapid fluctuations in its conformation and seems to play an important role in flap opening and closing.

### Docking studies with HIV‐1 PR inhibitors

**Figure 8:**
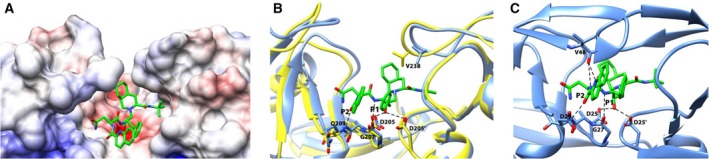
Docking of saquinavir with *Lm*Ddi1‐RVP. (A) Electrostatic potential surface diagram showing docked saquinavir (green) in the binding pocket. The pocket is hydrophobic except for an acidic patch formed by the catalytic aspartates that interact with the inhibitor. (B) Superimposition of the crystal structure (yellow) with the NMA model (blue) used for the docking. Polar interactions of the docked saquinavir with the protein are shown as dashed line. Saquinavir does not dock well with the crystal structure because the binding site is occluded by the flap resulting in steric clash of saquinavir with Val238. In the NMA model, flap moves outside positioning Val238 away from the binding pocket. (C) Characteristic interactions of saquinavir with HIV‐1.

Since our attempts to crystallize *Lm*Ddi1‐RVP in complex with saquinavir did not succeed, we carried out molecular docking studies to understand the possible nature of saquinavir binding. Docking of saquinavir with the crystal structure gave various binding poses, but none of these were close to the one seen in HIV‐1 PR in complex with saquinavir. Next, we used some of the models of the *Lm*Ddi1‐RVP domain obtained by NMA for the docking studies with saquinavir. The NMA models were energy minimized prior to docking. In one of the models, saquinavir docked in a similar manner as observed in the HIV‐1 PR complex, with a binding energy of −8.7 kcal·mol^−1^ (Fig. 8A). The results, on comparison with the crystal structure of HIV‐1 PR in complex with saquinavir (PDB Code: 3N3I), reveal that many critical interactions between the protein and the inhibitor are retained. In the docked structure, the OH group of saquinavir at the P1 position interacts with the side chains of the catalytic aspartates Asp205; the N atom at the P1 position makes a hydrogen bond with the carbonyl O atom of Gly207; and the O atom at the P3 position makes a hydrogen bond with the main chain N atom of Gln209 of *Lm*Ddi1‐RVP (Fig. 8B). All these interactions are observed in the crystal structure of HIV‐1 PR with saquinavir (Fig. 8C). The most significant difference between the two lies in the interaction of the flap with the inhibitor. The carbonyl O atom of Gly48 from the flap of HIV‐1 PR makes a hydrogen bond with the backbone amide N at the P2 position of the inhibitor. In the docked structure, the flap is too far from the inhibitor to have any interactions. The crystal structure does not dock well with saquinavir because one of the flaps in the crystal structure is closer to the active site, leading to steric clashes between the inhibitor and Val238 from the flap. In the NMA model, the flap moves outward positioning Val238 away from the binding pocket and allowing saquinavir to occupy the binding site (Fig. 8B). It is noteworthy that the NMA model has a slightly closed binding pocket compared to the crystal structure, which allows better interaction of the inhibitor with Gln209 and Gly207 residues.

### Structure comparison of *Lm*Ddi1‐RVP with *y*Ddi1‐RVP and *h*Ddi2‐RVP

**Figure 9:**
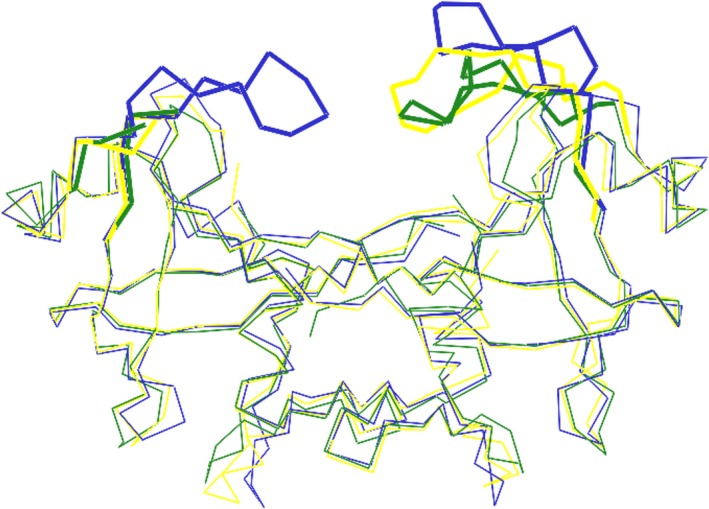
Comparison of RVP domain of Ddi1 from *Leishmania major*, yeast (PDB code: 4Z2Z) and human (PDB code: 4RGH). Superposition of *Lm*Ddi1‐RVP structure (blue) over *y*Ddi1‐RVP (green) and *h*Ddi2‐RVP subunit (yellow) showing conserved structure with main difference in the flap conformation. Flap density of only one subunit is present in *y*Ddi1‐RVP and *h*Ddi2‐RVP structure. The flap regions are highlighted in thicker lines.

**Figure 10:**
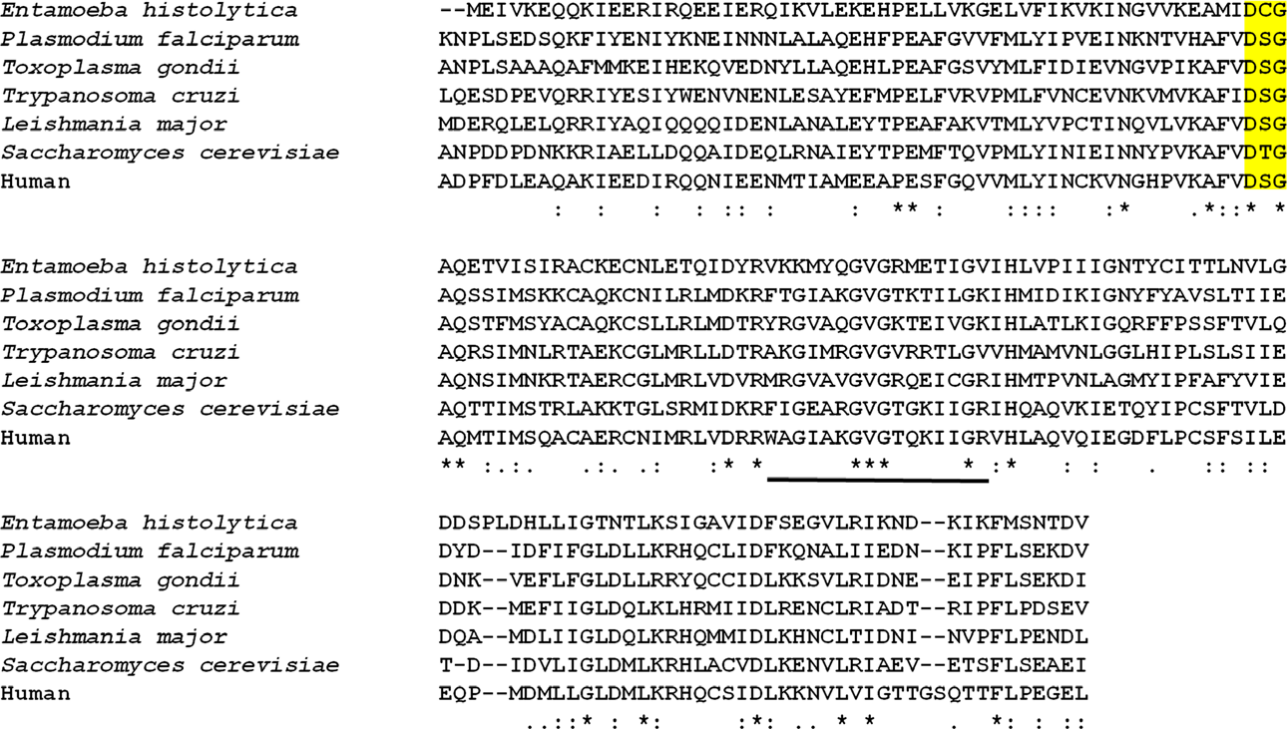
Sequence alignment of RVP domain of Ddi1 from different protozoans, yeast, and humans. The sequence shows very high similarity with catalytic motif DT/SG (highlighted in yellow) conserved. In *Entamoeba histolytica*, the S/T of the catalytic motif is replaced by a cysteine. The flap region of *Lm*Ddi1‐RVP is underlined.

The crystal structure information on the RVP domain of Ddi1 is available from *S. cerevisiae* (PDB Code: 2I1A, 4Z2Z) 27, 28 and human (PDB Code: 4RGH) proteins 29. The reported structures are similar to *Lm*Ddi1‐RVP, but the flap information is either missing or limited. In one of the yeast Ddi1 structures (PDB code: 2I1A), electron density for the flaps is completely missing, while in the other structure (PDB code: 4Z2Z), electron density for only one of the flaps is observed (Fig. 9). This flap is stabilized by making crystal contacts with the N‐terminal flexible linker region preceding the RVP domain. The linker inserts into the active site of an adjacent dimer acting as a pseudo‐substrate. It adopts an extended β‐sheet conformation and forms an antiparallel hydrogen bonding network with one part of the flap 28. In *h*Ddi2‐RVP also, only one flap that interacts with the symmetry‐related molecule has a well‐defined electron density 29. These observations suggest that the flap of the Ddi1‐RVP domain has a general characteristic of being more mobile and disordered compared to that of HIV‐1 PR and its density is observed only when its mobility is restricted due to crystal packing. The width of the binding cavity of *Lm*Ddi1‐RVP (23.8 Å) is similar to that of *h*Ddi2‐RVP (24.5 Å) and slightly different from that of *y*Ddi1‐RVP (26.0 Å). However, all the three protease domains have very similar overall structure. Even at the sequence level, they show very high similarity. The sequence for Ddi1‐RVP is highly conserved among different organisms (Fig. 10). The superimposition of individual subunits of Ddi1‐RVP from *L. major*,* S. cerevisiae*, and human suggests that the overall structure is very well conserved. But when one subunit of each Ddi1‐RVP dimer is superimposed, the second subunits deviate in position with RMSD values of 1.0–1.5 Å due to slightly differing angles (2.3–5.4°) between the two subunits in each dimer, suggesting a small relative movement of the subunits (Fig. 9).

## Discussion

HIV‐1 PR inhibitors cause a decline in protozoal infections in AIDS patients co‐infected with these pathogens. Ddi1‐RVP was shown to be the target of HIV‐1 PR inhibitors by White *et al*. 26. Lack of *in vitro* binding data with different inhibitors and poor understanding of the structure and dynamics of the protein have impeded the development of more effective inhibitors. Though the reported structures of *y*Ddi1‐RVP and *h*Ddi2‐RVP are close to *Lm*Ddi1‐RVP, the information on the flap in these structures is limited. Considering the fact that the flap of aspartic proteases plays a very important role in inhibitor/substrate binding, understanding of the structural arrangement and dynamics of the flap region in Ddi1‐RVP becomes important in determining the nature of binding. Further, HIV‐1 PR undergoes a significant movement upon substrate binding where the two subunits come closer and the flap closes over the substrate/inhibitor leading to a strong binding. However, it is still not clear whether the Ddi1‐RVP domain can undergo such a movement or not. If its structural framework makes it rigid, then it may not show strong binding with HIV‐1 PR inhibitors. The present study carried out on *Lm*Ddi1‐RVP provides insight into these aspects.

The crystal structure of *Lm*Ddi1‐RVP establishes the fact that the protein shares conserved structural features with HIV‐1 PR including the fold, the catalytic motif, and the hydrophobic substrate binding cavity. However, there are some notable differences between *Lm*Ddi1‐RVP and HIV‐1 PR. One is that the binding cavity of *Lm*Ddi1‐RVP is larger compared to that of HIV‐1 PR. A similar feature has also been observed in the case of *y*Ddi1‐RVP 27. Also, there are variations in the binding site residues. The S1/S1′ subsite is hydrophobic but contains a bulkier group Phe203 as compared to HIV‐PR. Also, S3/S3′ is more hydrophobic due to the presence of Met188 and Met271 residues. Instead of Asp29, Asp30, and Val82, which interact with inhibitors in HIV‐1 PR, *Lm*Ddi1‐RVP has Gln209, Asn210, and Asp272, respectively. These variations change the nature of the surface charge and polarity, and may alter the binding of the inhibitor. Further, the flap in HIV‐1 PR assumes defined secondary structural features, forming a β‐hairpin structure in the open and the closed forms. Contrary to this, the flap of *Lm*Ddi1‐RVP, though similar in length to that of HIV‐1 PR, does not assume any defined secondary structure and remains very mobile. Due to its high mobility, the density is generally missing as has also been observed in the structures of *y*Ddi1‐RVP and *h*Ddi2‐RVP as well as in *Lm*Ddi1‐RVP that crystallized in the P1 space group. The electron density for the flaps of both the subunits is visible in *Lm*Ddi1‐RVP in the P2_1_ space group because it interacts with the other molecule of the asymmetric unit and hence becomes restricted in its movement. High mobility of the flaps seems to be a characteristic feature of Ddi1‐RVP. Even upon binding to a symmetry‐related molecule, it does not acquire a β‐hairpin structure, as is evident in the ordered flap region of *y*Ddi1‐RVP 28. The larger binding cavity and the presence of such a flexible flap suggest that Ddi1‐RVP may accommodate bigger substrates or may act on more than one substrate. In a recent study, Nrf1 was proposed to be the natural substrate of *h*Ddi2; but no homolog of Nrf1 is reported in *L. major* or in other protozoans. This suggests that either the corresponding substrate of the *Lm*Ddi1‐RVP has a very different sequence compared to its *S. cerevisiae* and human counterparts, or *Lm*Ddi1‐RVP acts on altogether different target protein(s).

Further, we carried out NMA to look for the global concerted motion of the protein to explore the possibility of modulation of the size of the binding pocket. NMA analysis reveals that *Lm*Ddi1‐RVP has structural flexibility similar to that of HIV‐1 PR and may undergo similar movement where the two domains can come closer along with the closure of the flap upon substrate/inhibitor binding. Docking of saquinavir with the crystal structure and NMA model of *Lm*Ddi‐RVP suggests that binding may require/induce some conformational changes in the protein, since the NMA model showed better binding than the crystal structure. Overall binding seems to be similar to that found in the HIV‐1 PR–saquinavir complex but may require some changes from the crystal structure of *Lm*Ddi‐RVP to bring the subunits closer and open up the flap for better access of saquinavir to the binding pocket.

The affinity of *Lm*Ddi‐RVP for HIV‐1 PR inhibitors is in micromolar range (compared to nanomolar or lower values for HIV‐1 PR) in solution as suggested by BLI studies. The presence of a very mobile flap having a different sequence compared to that of HIV‐1 PR may result in weaker binding in *Lm*Ddi‐RVP. In HIV‐1 PR, the inhibitors interact with the flap and any mutation in the flap affects the binding. The reason for diminished or lack of binding can be attributed to a loss of interaction or change in the flap dynamics with a change in sequence. MD simulation studies that we carried out on *Lm*Ddi1‐RVP reveal that the flap conformation in the crystal structure is a result of crystal packing, and the preferred state is a semi‐open one like that found in HIV‐1 PR. The flap is highly flexible and does not assume a β‐hairpin structure during the course of 300 ns simulation, even in the semi‐open state. The flap movement does not perturb other parts of the protein, unlike in HIV‐1 PR where it is accompanied by significant concerted movement in the flap elbow and other regions of the protein resulting in tight closure of the active site over the inhibitor in the bound state. The independent movement of the flap in *Lm*Ddi1‐RVP seems to be due to additional secondary structural elements in the flap elbow region that prevent the effect of flap movement from being transmitted to other parts of the protein. Unlike in HIV‐1 PR, where flap behavior is more symmetric, the structural fluctuations in *Lm*Ddi1‐RVP are asymmetric. One flap shows more mobility, while the other is not only less mobile but also moves close to the active site.

Development of effective inhibitors against Ddi1‐RVP will require a detailed understanding of the structure of the protein in complex with the inhibitors. Also, a better understanding of structural differences of this protein from different protozoans will be required for creating a strong structural base for structure‐guided inhibitor development. Our efforts are underway to determine the structures of Ddi1 from different opportunistic pathogens and also to co‐crystallize the protein with various inhibitors. As mentioned before, the interacting segment of the flap can be used as a starting point to design more specific peptide inhibitors for the Ddi1‐RVP domain. Current work will act as a primer in the structural understanding of Ddi1‐RVP from pathogenic protozoans that will assist in devising a proper strategy for the development of effective inhibitors.

Efforts toward the development of drugs against protozoal infections, exploiting the observed ability of HIV‐PR inhibitors to be effective against opportunistic infections, were impeded initially as the target was unknown. Though subsequent studies indicated Ddi1, which is also an aspartic protease, as a possible target, lack of complete structural details and direct binding studies slowed down the process significantly. Our present study reporting the crystal structure of *Lm*Ddi1, the only aspartic protease present in the organism, provides a structural platform for further work, and the binding affinity shown between *Lm*Ddi1 and one of the inhibitors, though not very strong, will lead to further investigations on Ddi1 in other organisms.

## Author contributions

KS and SK designed the project. SK carried out the experiments. KS and SK analyzed the results and wrote the manuscript.
